# Counting the cost of premature mortality with progressively worse aortic stenosis in Australia: a clinical cohort study

**DOI:** 10.1016/S2666-7568(22)00168-4

**Published:** 2022-08-18

**Authors:** Simon Stewart, Clifford Afoakwah, Yih-Kai Chan, Jordan B Strom, David Playford, Geoffrey A Strange

**Affiliations:** Institute for Health Research, University of Notre Dame Australia, Fremantle, WA, Australia; School of Medicine, Dentistry & Nursing, University of Glasgow, Glasgow, UK; Menzies Health Institute Queensland, Griffith University, Gold Coast, QLD, Australia; Centre for Applied Health Economics, Griffith University, Brisbane, QLD, Australia; Menzies Health Institute Queensland, Griffith University, Gold Coast, QLD, Australia; Torrens University Australia, Adelaide, SA, Australia; Mary MacKillop Institute for Health Research, Australian Catholic University, Melbourne, VIC, Australia; Richard A And Susan F Smith Centre for Outcomes Research in Cardiology, Beth Israel Deaconess Medical Centre, Boston, MA, USA; Harvard Medical School, Boston, MA, USA; Institute for Health Research, University of Notre Dame Australia, Fremantle, WA, Australia; Institute for Health Research, University of Notre Dame Australia, Fremantle, WA, Australia; Faculty of Medicine and Health, The University of Sydney, Sydney, NSW, Australia

## Abstract

**Background:**

Aortic stenosis is the most common cardiac valve disorder requiring clinical management. However, there is little evidence on the societal cost of progressive aortic stenosis. We sought to quantify the societal burden of premature mortality associated with progressively worse aortic stenosis.

**Methods:**

In this observational clinical cohort study, we examined echocardiograms on native aortic valves of 98 565 men and 99 357 women aged 65 years or older across 23 sites in Australia, from Jan 1, 2003, to Dec 31, 2017. Individuals were grouped according to their peak aortic valve velocity in 0·50 m/s increments up to 4·00 m/s or more (severe aortic stenosis), using 1·00–1·99 m/s (no aortic stenosis) as the reference group. Sex-specific premature mortality and years of life lost during a 5-year follow-up were calculated, along with willingness-to-pay to regain quality-adjusted life years (QALYs).

**Findings:**

Overall, 20 701 (21·0%) men and 18 576 (18·7%) women had evidence of mild-to-severe aortic stenosis. The actual 5-year mortality in men with normal aortic valves was 32·1% and in women was 26·1%, increasing to 40·9% (mild aortic stenosis) and 52·2% (severe aortic stenosis) in men and to 35·9% (mild aortic stenosis) and 55·3% (severe aortic stenosis) in women. Overall, the estimated societal cost of premature mortality associated with aortic stenosis was AU$629 million in men and $735 million in women. Per 1000 men and women investigated, aortic stenosis was associated with eight more premature deaths in men resulting in 32·5 more QALYs lost (societal cost of $1·40 million) and 12 more premature deaths in women resulting in 57·5 more QALYs lost (societal cost of $2·48 million) when compared with those without aortic stenosis.

**Interpretation:**

Any degree of aortic stenosis in older individuals is associated with premature mortality and QALYs. In this context, there is a crucial need for cost-effective strategies to promptly detect and optimally manage this common condition within our ageing populations.

**Funding:**

Edwards LifeSciences, National Health and Medical Research Council of Australia, and the National Heart, Lung, and Blood Institute.

## Introduction

As individuals living in high-income countries increasingly benefit from preventive heart health measures to prolong their life, they become more likely to develop age-related heart conditions. Paradoxically, having avoided early and often fatal forms of heart disease, these individuals might still be at risk of dying prematurely.^1^ One such condition affecting the aortic valve is aortic stenosis, which is the most common form of valvular heart disease treated in clinical practice and is a major cause of death among those aged 75 years or older.^2^ In the context of a high prevalence of cardiac risk factors (including hypertension and metabolic disease^3^), data suggest that around 1·5% of people aged 55 years or older (with a steep age-gradient in rising prevalence) in high-income countries are already living with severe aortic stenosis.^4,5^

Traditionally, surgical aortic valve replacement is reserved for patients with severe symptoms.^6^ The development of less invasive, transcatheter techniques for aortic valve replacement has altered the benefit-to-risk ratio of aortic stenosis management and assessment of treatment safety and efficacy is ongoing in patients with less severe symptoms.^7^ Regardless of treatment modality, the increasingly prevalent condition of aortic stenosis poses an enormous challenge to already limited health-care resources.^8^ Crucially, there is increasing evidence showing that less severe forms of aortic stenosis are associated with premature mortality, whether by causation or correlation.^9^ However, there remains minimal evidence of the societal cost of progressive aortic valve disease.^10^

Therefore, in this study, we firstly aimed to identify the sex-specific distribution of progressive aortic stenosis among older individuals being routinely investigated for heart disease. We then determined the association of progressive aortic stenosis with quality-adjusted life years (QALYs) lost due to premature mortality, compared with individuals presenting with normal aortic valve function.

## Methods

### Study design and population

We conducted an observational clinical cohort study based on the previously described National Echo Database of Australia (NEDA),^11^ using the Reporting of Studies Conducted Using Observational Routinely Collected Health Data guidelines.^12^ NEDA has obtained ethical approvals across Australia from all relevant institutional, university and government Human Research Ethics Committees (HREC). A patient consent waiver has been authorised by each HREC for retrospective data, and prospective consent is obtained by verbal script at each centre using an opt-out process. The NEDA cohort comprises individuals aged 65 years or older with native aortic valves who received echocardiograms investigating aortic stenosis severity (with a peak aortic valve velocity [Vmax] of 1·0 m/s or more on the last recorded echocardiogram) across 23 centres in Australia from Jan 1, 2003, to Dec 31, 2017 (appendix p 2). We excluded patients with severe haemodynamic compromise of their aortic valve (Vmax <1·0 m/s). We also excluded patients whose native aortic valve had been surgically replaced during follow-up. As previously described,^11^ NEDA independently extracts (thereby minimising bias) the age, sex, anthropometric profile, and a standard list of left and right heart parameters of patients being routinely assessed by echocardiography. All identified patients were individually linked from the time of their last echocardiogram to the well validated National Death Index^13^ to determine all-cause mortality up to and including Dec 31, 2018.

### Outcomes

The primary outcomes of this study were years of life lost (YLL) and the associated societal costs attributable to premature mortality during a fixed 5-year follow-up from last echocardiogram according to an individual’s baseline aortic valve function (from normal to severe aortic stenosis). For all outcome analyses, Vmax (which increases linearly with aortic stenosis severity) recorded on the last echocardiogram was aggregated into six prospectively selected groups. The group with the lowest Vmax values Vmax (indicating no aortic stenosis) was defined as the reference group for all subsequent analyses. The prospectively selected groups were: Vmax 1·00–1·99 m/s (no aortic stenosis or reference), 2·00–2·49 m/s (mild aortic stenosis), 2·50–2·99 m/s (mild-to-moderate aortic stenosis), 3·00–3·49 m/s (moderate aortic stenosis), 3·50–3·99 m/s (moderate-to-severe aortic stenosis), and 4·00 m/s or higher (severe aortic stenosis).^14,15^ The proportion of men and women categorised within each group was then calculated.

Age at death was then used to identify premature mortality based on sex-specific life-expectancy thresholds in Australia in 2020 (80·7 years for men and 84·9 years for women).^16^ For each Vmax group, the number of YLL during complete 5-year follow-up was calculated. To examine the societal burden of premature mortality according to progressive aortic stenosis (relative to the no aortic stenosis group) we calculated: (1) the total QALYs lost in each group during 5-year follow-up, and (2) willingness-to-pay to regain each QALY if there were the capacity to restore equivalent levels of premature mortality and associated YLL to those observed in the no aortic stenosis group. For these estimates we used data from Lee and colleagues,^17^ who estimated that the quality of life (using the EQ-5D-3L questionnaire) in people with severe aortic stenosis who were receiving transcatheter aortic valve implantation to be 0·71 at baseline (mean age of 86 years). With no equivalent estimates for those living with mild-to-moderate forms of the condition (largely because they do not typically receive interventions), for our main analyses we applied the same quality of life levels to the remaining, non-severe aortic stenosis groups.

### Statistical analysis

Given the substantive size of the patient cohort being studied and our intention not to formally compare groups and outcomes, no formal power calculations of the sample size needed were conducted. The sex-specific distribution of progressive aortic stenosis according to Vmax and the associated pattern of actual 5-year mortality (with no imputation of data) was firstly described for the entire NEDA cohort. For each group, the number of all-cause deaths, premature deaths and associated YLL and QALYs were calculated based on actual person-years accumulated during a fixed 5-year follow-up. QALY was calculated as a product of YLL and quality of life value. For comparative purposes, these data were then converted into the distribution of Vmax groups and proportional outcomes per 1000 men and 1000 women undergoing echocardiographic investigation. All subsequent comparisons are described (without inferential statistical analyses) as the excess number of events and costs (from a societal perspective) relative to the reference group. Huang and colleagues^18^ estimated that one QALY was worth AU$42 000 over a 2-year rolling window using Australian population-based data. Cost analysis was done from a societal perspective and results are reported in 2020 values after adjusting for inflation using the consumer price index from the Australian Bureau of Statistics and 2018 price level as the base. An inflation-adjusted willingness-to-pay of $43 038 was used for our baseline cost analysis. Currently, NEDA does not capture detailed clinical data (including underlying risk factors, comorbidity, and pharmacological therapy) and therefore the reported pattern of mortality within each discrete study group has not been adjusted for these potential confounders.

We performed three sensitivity analyses. First, we relaxed the assumption that patients with mild-to-moderate aortic stenosis have the same quality of life (utility score of 0·71) as patients with severe aortic stenosis, replacing this value with a recent estimate for chronically ill patients in Australia (utility score of 0·79).^19^ Second, we applied a quality-of-life utility score of 0·55 derived from a US cohort based on Kansas City Cardiomyopathy Questionnaire scores for patients with intermediate-risk aortic stenosis receiving the next-generation SAPIEN 3 transcatheter heart valve intervention.^20^ Together, these values provide upper and lower bound estimates for our baseline QALY estimates. Thirdly, we applied a higher cost per QALY ($68 675) based on a 5-year rolling window using Australian population-based data to assess long-term willingness-to-pay.^18^

Confidence intervals were calculated using the standard formula:

$$P=proportion\pm 1.96\sqrt{\frac{proportion(1-proportion)}{n}}$$

where, the proportion,

$$\sqrt{\frac{proportion(1-proportion)}{n}}$$

is standard deviation of the sample distribution, and n is sample size for each sex group.

Data were prepared and analysed using SPSS version 26.0 and STATA version 13.0.

### Role of the funding source

The funders of this study had no role in study design, data collection, data analysis, data interpretation, or writing of the report.

## Results

**Figure 1:**
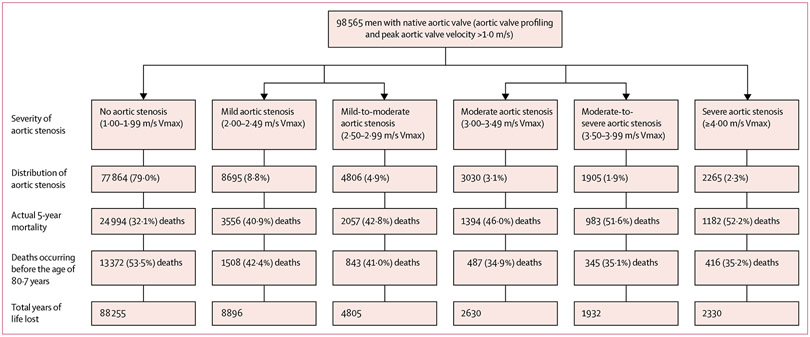
Profile of progressively worse aortic stenosis and associated 5-year all-cause mortality in men This figure shows the proportion of men in each Vmax group (second level), and mortality outcomes based on the number who died during 5-year follow-up (third level), the proportion of premature deaths occurring before the age of 80·7 years (fourth level) and the associated total and mean years of life lost (fifth level) including 95% CI. Median (interquartile) years of follow-up from last echocardiogram to study completion was 6·1 (IQR 3·9–10·2) for no aortic stenosis, 6·4 (4·0–10·2) for mild aortic stenosis, 6·8 (4·2–10·6) for mild-to-moderate aortic stenosis, 7·4 (4·5–10·9) for moderate aortic stenosis, 7·6 (4·7–11·2) for moderate-to-severe aortic stenosis, and 8·7 (5·3–11·7) for severe aortic stenosis years. Vmax=peak aortic valve velocity. YLL=years of life lost.

**Figure 2:**
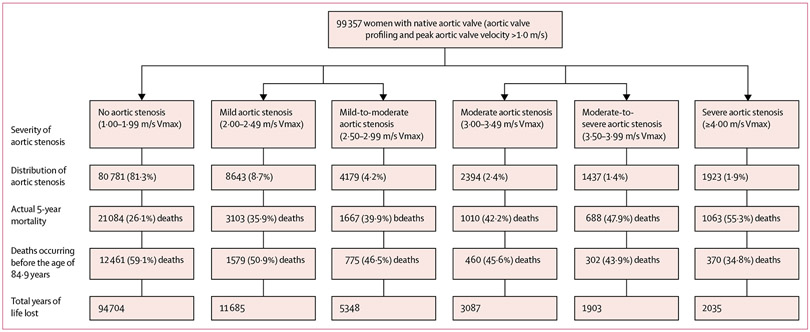
Profile of progressively worse aortic stenosis and associated 5-year all-cause mortality in women This figure shows the proportion of women in each Vmax group (second level), and mortality outcomes based on the number who died during 5-year follow-up (third level), the proportion of premature deaths occurring before the age 84·9 years (fourth level) and the associated total and mean YLL (fifth level) including 95% CI. Median (interquartile) years of follow-up from last echocardiogram to study completion was 6·1 (3·9–10·0) for no aortic stenosis, 6·4 (4·0–10·1) for mild aortic stenosis, 6·8 (4·9–10·5) for mild-to-moderate aortic stenosis, 7·5 (4·6–10·8) for moderate aortic stenosis, 7·9 (4·9–11·1) for moderate-to-severe aortic stenosis, and 7·8 (4·8–11·1) for severe aortic stenosis. Vmax=peak aortic valve velocity. YLL=years of life lost.

Of the 217 599 participants aged 65 years or older with a native aortic valve (appendix p 2), we included 98 565 men and 99 357 women from the NEDA cohort (representing 31·3% of all 631 824 participants) aged 65 years or older. Among those excluded from analyses, 3474 were men and 1980 were women whose native aortic valve had been surgically replaced after their first echocardiogram. We also excluded 8698 men and 5015 women with severe haemodynamic compromise of their native aortic valve (Vmax <1·0 m/s; appendix p 2). A total of 127 807 individuals had complete 5 years of follow-up, during which 40 890 (32·0%) died. Within the cohort, 77 864 of 96 565 men (79·0%, 95% CI 78·7–79·3), aged 74·7 years (SD 6·9), had no aortic stenosis at baseline. A similar pattern was observed in the slightly older cohort of women, of whom 80 781 of 99 357 (81·3%, 95% CI 81·1–81·6), aged 76·0 years (7·4), had no aortic stenosis at baseline. In men, progressively worse aortic stenosis was associated with increasing age (range 77·1 [SD 7·2] to 79·0 [7·2] years; table). Overall, in men, 8·8% (95% CI 8·7–9·0) had mild aortic stenosis, 4·9% (4·7–5·0) had mild-to-moderate aortic stenosis, 3·1% (3·0–3·2) had moderate aortic stenosis, 1·9% (1·9–2·0) had moderate-to-severe aortic stenosis, and 2·3% (2·2–2·4) had severe aortic stenosis (figure 1). In women, progressively worse aortic stenosis was also associated with increasing age (range 78·4 [SD 7·6] to 81·8 [7·6] years; table). Overall, in women, 8·7% (95% CI 8·5–8·9) had mild aortic stenosis, 4·2% (4·1–4·3) had mild-to-moderate aortic stenosis, 2·4% (CI 2·3–2·5) had moderate aortic stenosis, 1·5% (1·4–1·5) had moderate-to-severe aortic stenosis, and 1·9% (1·9–2·0) had severe aortic stenosis (figure 2).

Actual 5-year mortality increased with progressively higher Vmax levels in both men (from 32·1% in those with no aortic stenosis to 52·2% in those with severe aortic stenosis; figure 1) and women (from 26·1% in those with no aortic stenosis to 55·3% in those with severe aortic stenosis; figure 2). Conversely, reflecting the increasing age of individuals presenting with more severe forms of aortic stenosis, the proportion of premature deaths declined in men from 53·5% in the mild aortic stenosis group to 35·2% in the severe aortic stenosis group and in women from 59·1% in the mild aortic stenosis group to 34·8% in the severe aortic stenosis group. If a premature death did occur, it was more likely to be categorised as cardiovascular-related with progressively worse aortic stenosis in both sexes (appendix p 3).

**Figure 3:**
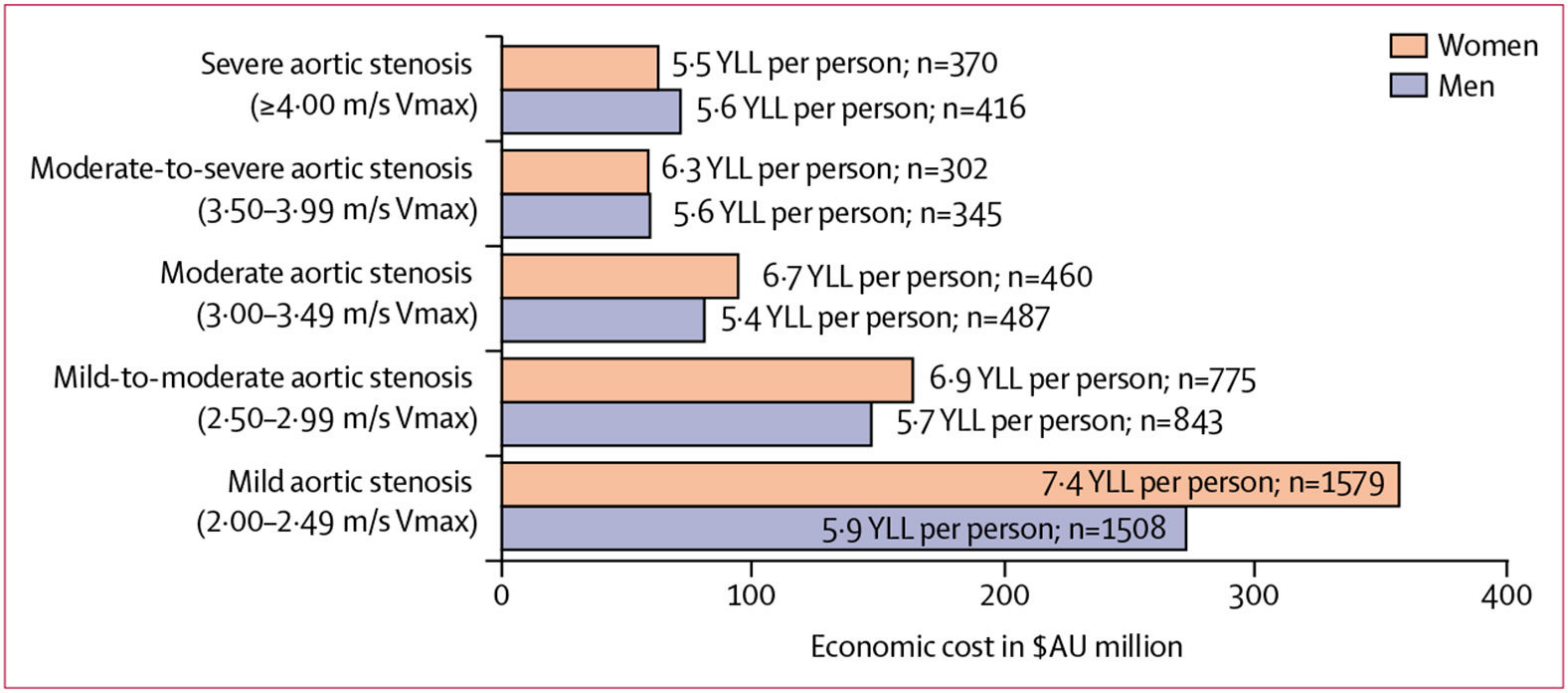
Sex-specific cost of premature mortality in cases with any form of aortic stenosis (n=39 277) This figure shows the economic cost of premature mortality (based on willingness-to-pay per quality adjusted life-year) during fixed 5-year follow-up according to progressively worse aortic stenosis observed within the National Echo Database of Australia cohort. The mean number of YLL per person and total premature deaths within each group is also presented. Vmax=peak aortic valve velocity. YLL=years of life lost.

Overall, the estimated societal cost of premature mortality observed within the entire cohort was $6·29 billion. With a mean of 6·6 YLL (SD 4·3) per death in men and 7·6 YLL (5·2) per death in women, the societal cost of premature mortality (25 833 deaths) over the 5-year follow-up within the no aortic stenosis (reference) group was $2·70 billion in men and $2·89 billion in women. The total equivalent cost of premature mortality and associated YLL among individuals with any form of aortic stenosis, despite their advanced age, was also substantive ($629 million from 20 593 premature deaths in men and $735 million from 24 058 premature deaths in women). Due to a combination of more premature deaths overall and greater YLL per death, societal costs were higher among men and women with mild-to-moderate forms of aortic stenosis than among those with more severe forms of aortic stenosis (figure 3). Among men, the short-term societal cost (using willingness-to-pay $43 038 per QALY) of the excess eight premature deaths and 32·5 more QALYs lost associated with all cases of aortic stenosis detected per 1000 echocardiograms was $1·40 million, when compared with those without aortic stenosis. Among women, the equivalent societal cost of the excess 12 premature deaths and 57·5 more QALYs lost associated with all cases of aortic stenosis detected per 1000 echocardiograms was $2·48 million (table).

Sensitivity analyses (appendix p 4) revealed that when an upper bound quality of life value of 0·79 was applied to the YLL estimates in the table, any form of aortic stenosis was associated with an estimated 35·7 QALYs lost per 1000 men and 63·1 QALYs lost per 1000 women. Accordingly, the estimated societal cost rose to $1·54 million for men and $2·72 million for women. Alternatively, when applying a lower bound quality of life value of 0·55, the estimated QALYs lost per 1000 men was 31·5 and per 1000 women was 43·5. Thus, the estimated societal cost was reduced to $1·35 million for men and $1·87 million for women. Finally, when applying our original quality of life value of 0·71 and willingness-to-pay for long-term health gains ($68 675 per QALY), the overall estimated societal cost of progressively worse aortic stenosis was $2·2 million per 1000 men and $3·9 million per 1000 women.

## Discussion

To our knowledge, this represents the first study to examine the sex-specific pattern of premature mortality and subsequent YLL associated with progressive aortic stenosis in a large, real-world cohort of individuals being routinely investigated for heart disease. Due to a combination of factors, including the age profile of those presenting with less severe forms of aortic stenosis, we identified that the greatest societal burden attributable to premature mortality is not confined to those presenting with severe aortic stenosis. As expected, patients with severe aortic stenosis (comprising 1·9% of women and 2·3% of men) had typically high 5-year mortality and according to current guidelines^15^ would qualify for an aortic valve replacement if also symptomatic (noting the health constraints in delivering such an intervention).^21^ However, we also found that among those individuals presenting with mild-to-moderate aortic stenosis (affecting 18·7% of men and 16·8% of women), levels of premature mortality and associated QALYs lost within 5 years were substantially high. Despite a similar distribution of aortic stenosis, overall, women had a greater number of YLL, QALYs lost, and societal costs than men due to their higher expected longevity. Similarly, the relative high impact of mild-to-moderate forms of aortic stenosis on both premature mortality and QALYs reflected the dynamics of three key factors: (1) the larger numbers of women and men with mild-to-moderate forms (compared with those with severe aortic stenosis), (2) their younger age profile, meaning more potential for premature mortality (particularly for women, given the higher threshold for a premature death) and, (3) increasingly higher 5-year mortality in individuals with any form of aortic stenosis (compared with those with no aortic stenosis). Within the mild-to-moderate aortic stenosis cohort, who typically remain untreated based on current guidelines,^15^ the societal cost of premature mortality was estimated to be $1·40 million per 1000 men and $2·48 million per 1000 women, over and above that observed in the no aortic stenosis group. Even when considering the potentially lower estimates derived from sensitivity analyses, these findings suggest that patients with non-severe forms of aortic stenosis still have a high societal burden, either by causation or correlation.

Unfortunately, there is a scarcity of evidence to support the prevention of this common condition.^15^ For example, despite their proven efficacy for other forms of heart disease, statins do not slow progression of aortic stenosis;^22^ which can be rapid in some individuals.^23,24^ The same applies to other cardiac prevention strategies. As a direct therapeutic measure, both surgical^6^ and transcatheter^7^ forms of aortic valve replacement are proven strategies to prolong survival in severe cases of aortic stenosis, particularly when normalised Vmax levels are reached.^25^ However, as highlighted by a 2022 analysis of the caseload of severe cases in the UK (an estimated 1·45% of the population aged 55 years or older),^5^ there is limited scope for the National Health Service to scale-up surgical services.^26^

Even in the most well resourced health systems, aortic valve replacement rates remain lower than expected.^27^ The inexorable ageing of many high-income populations will inevitably impart an increasingly challenging and unsustainable societal burden and health-care expenditure if there is no development and application of pragmatic preventive strategies for key contributors of future costs such as aortic stenosis.

Even if aortic stenosis cannot be effectively managed directly without resorting to resource-intensive and costly surgical options, the evidence-based treatment of concurrent heart failure has been transformed in recent years with positive results from clinical trials of angiotensin receptor-neprilysin inhibitors and SGLT2 inhibitors to supplement the proven benefits of other neurohormonal antagonists.^28^ The positive results from clinical trials are clinically relevant and important given that in a parallel cohort of US patients matched with the same Vmax levels of those in the NEDA cohort, we found the proportion of individuals concurrently presenting and being treated for heart failure progressively rose from 38·6% in men and 36·8% in women, among individuals with no aortic stenosis, to 59·5% in men and 65·7% in women, among those presenting with severe aortic stenosis.^29^ The SGLT2 inhibitors appear to convey benefits across the entire spectrum of heart failure. As recently shown within the NEDA cohort, any decline in ejection fraction is of prognostic importance in many individuals.^30^ Given the high cost of health-care expenditure for patients with heart failure and the proven cost-effectiveness of readily available medical therapy,^28^ there is a compelling argument for alerting an individual’s health-care team of the need for more proactive surveillance and management (including evidence-based heart failure treatments) once mild-to-moderate aortic stenosis has been detected.

The dynamics of sex-specific, premature mortality vary from country to country as do the costs of managing common forms of chronic heart disease. According to the Organisation for Economic Co-operation and Development (OECD), Australia is one of the top five countries for longevity for both men and women.^31^ The calculated proportion of premature deaths and associated YLL would be lower for countries such as the UK where life expectancy is reportedly 2–4 years lower.^5^ This same heterogeneity would apply to each society’s willingness-to-pay for each QALY lost, but our findings reinforce a greater focus on quantifying the quality of life of those affected by mild-to-moderate forms of aortic stenosis. Given the close association between progressively worse aortic valve disease and advancing age (the mean age individuals found to have severe stenosis of their native aortic valve was 79 years for men and 82 years for women), there is a potential argument that this condition is an inevitable consequence of ageing populations that now survive previously fatal events at a younger age.^1^ Although this argument might apply to a good proportion of individuals who exceeded average life expectancy in Australia (around two-thirds with severe aortic stenosis within our cohort), there was still a large component of individuals (around half of those with mild aortic stenosis) who did not. By any measure, the current societal cost of progressive aortic stenosis is substantive, and this will undoubtedly translate into substantive health-care costs. Compared with other OECD countries, Australia typically spends more on health care and provides core services to the majority of the population.^31^ However, Australia currently lags with regards to the provision of primary care services and preventive health services, and these might be the most important investments to tackle an evolving burden of heart disease.^31^

We purposefully focused on individuals aged 65 years or older within the NEDA cohort who had a Vmax level measured across their native aortic valve given that this is the age bracket in which aortic stenosis is most common. We also excluded individuals with severely compromised cardiac function who typically have high mortality rates owing to their advanced heart disease.^11^ Although we report on actual mortality and standardised premature mortality or YLL in a very large clinical cohort, by necessity, we did have to apply several assumptions. Firstly, we cannot exclude the possibility that some individuals did undergo surgical replacement of their aortic valve following their last echocardiogram. Secondly, we assumed that mortality rates across the Vmax groups remained the same in participants of the NEDA cohort with later echocardiograms (ie, individuals with insufficient follow-up to calculate 5-year mortality). Although previous analyses have shown improving survival rates across historical epochs within the NEDA cohort,^11,30^ there is no evidence to suggest that these improved survival rates favoured those with aortic stenosis. Despite the greater number of individuals with non-severe aortic stenosis, compared with those with severe forms, there remains a paucity of data on the quality of life of individuals with non-severe forms. We therefore assumed indices of QALY and subsequent willingness-to-pay to regain QALY lost were largely static across the aortic stenosis groups. Our sensitivity analyses revealed consistent and substantial costs of untreated mild-to-moderate aortic stenosis. Our cost estimates are very useful as baseline costs for evaluating the cost-effectiveness of health-care interventions that seek to improve the longterm survivorship of patients with mild-to-moderate aortic stenosis. Because NEDA has yet to capture detailed clinical data, we report on the association between varying levels of aortic stenosis and mortality, rather than establishing causality. We also did not undertake any adjusted analyses to determine if progressive aortic stenosis independently contributes to the observed pattern of increasing mortality. However, we recently completed two collaborative studies with equivalent Northern American^29^ and European^32^ cohorts with detailed clinical phenotyping. In both analyses, direct comparisons between these cohorts and the NEDA cohort showed similar patterns of increasing mortality with the same unit increases in Vmax levels.^29,32^ Moreover, in both these studies, increasing Vmax levels remained a strong and independent prognostic marker for increasing mortality after extensive adjustment for potential clinical confounders.^29,32^ NEDA also does not capture data on the race and ethnicity of individuals, which have been shown to be important predictors of premature mortality in other health systems (though, to note, Australia provides universal health coverage).^33^ Beyond replicating our broad findings in other large clinical cohorts, future research is needed to better delineate the clinical trajectory and cause of death of individuals affected by mild-to-moderate forms of the disease.^9-11,24^

Although we report on the pattern of premature mortality and YLL associated with progressively worse aortic stenosis within an Australian clinical cohort, our findings are broadly generalisable to other countries with a large burden of disease imposed by aortic stenosis, including the UK^5^ and countries in continental Europe and North America.^4^ The individual and societal imperative to more readily detect and clinically manage less severe forms of this common clinical condition to reduce the societal cost of future premature mortality, as well as a probable high burden of related health expenditure, is likely to apply to any country with substantial population ageing.

## Supplementary Material

1

## Figures and Tables

**Table:** Aortic valve profile and subsequent pattern of mortality per 1000 men and 1000 women

	No aortic stenosis (1·00–1·99 m/s Vmax)	Mild aortic stenosis (2·00–2·49 m/s Vmax)	Mild-to- moderate aortic stenosis (2·50–2·99 m/s Vmax)	Moderate aortic stenosis (3·00–3·49 m/s Vmax)	Moderate-to- severe aortic stenosis (3·50–3·99 m/s ^Vmax)^	Severe aortic stenosis (≥4·00 m/s Vmax)
**Per 1000 men with aortic valve profiling (echocardiogram)**						
Distribution of cases	790	88	49	31	19	23
Mean baseline age, years	74·7 (6·9)	77·1 (7·2)	77·8 (7·2)	78·4 (7·2)	78·7 (7·3)	79·0 (7·2)
Actual 5-year mortality	254 (32·1%)	36 (40·9%)	19 (42·8%)	13 (46·0%)	9 (51·6%)	11 (52·2%)
Premature deaths occurring before the age of 80·7 years	136 (53·5%)	15 (42·4%)	8 (41·0%)	5 (34·9%)	3 (35·1%)	4 (35·2%)
Excess deaths	Reference group	8	5	4	4	4
Excess premature deaths	Reference group	3	2	1	1	1
Excess QALY	Reference group	12·6	8·1	3·8	4·0	4·0
Willingness-to-pay for lost QALY ($AU)	Reference group	542 000	349 000	164 000	172 000	172 000
**Per 1000 women with aortic valve profiling (echocardiogram)**						
Distribution of cases	813	87	42	24	15	19
Mean baseline age, years	76·0 (7·5)	78·4 (7·6)	79·6 (7·5)	79·9 (7·7)	80·5 (7·4)	81·8 (7·6)
Actual 5-year mortality	212 (26·1%)	31 (35·9%)	17 (39·9%)	10 (42·2%)	7 (47·9%)	11 (55·3%)
Premature deaths occurring before the age of 84·9 years	125 (59·1%)	16 (50·9%)	8 (46·5%)	4 (45·6%)	3 (43·9%)	4 (34·8%)
Excess deaths	Reference group	9	6	4	3	6
Excess premature deaths	Reference group	4	3	2	1	2
Excess QALY	Reference group	21·0	14·7	9·5	4·5	7·8
Willingness-to-pay for lost QALY ($AU)	Reference group	904 000	633 000	409 000	194 000	336 000

Data are n, mean (SD) or n (%) unless otherwise specified. This table summarises the additional societal cost of premature mortality associated with progressively worse aortic stenosis, relative to those presenting with no aortic stenosis per 1000 men and per 1000 women aged 65 years or older and with profiling of their aortic valve function within the National Echo Database of Australia cohort. The number of calculated individuals affected and resulting deaths or premature deaths are rounded up into whole numbers. QALY=quality adjusted life-year. Vmax=peak aortic valve velocity.

## Data Availability

Requests for anonymised aggregated cohort data can be submitted to the corresponding author for institution consideration. Other datasets such as the health-related quality of life and the monetary value of health are all available from public sources.^17-19^

## Research in context

### Evidence before this study

Aortic stenosis is the most common valvular disorder requiring clinical management and is closely linked to advancing age. When left undetected or untreated, it is associated with high morbidity and mortality, even amongst those with mild disease. We performed a comprehensive search of PubMed to identify all relevant articles on the cost impact of aortic stenosis published in the English language from Jan 1, 2000, to March 1, 2022. The search strategy was based on the combination of relevant terms and names including “aortic stenosis”, “aortic valve disease”, “premature mortality”, “mortality”, “quality-adjusted life years”, and “years of life lost”. Currently, there is little real-word evidence to quantify the broad impact that aortic stenosis has among those living (and dying) with this increasingly common condition.

### Added value of this study

To our knowledge, this is one of the first large-scale clinical cohort studies assessing the association of progressive aortic stenosis and subsequent patterns of premature mortality and years of life lost over a fixed time-period (5 years). With the capture of routine echocardiographic profiling data on all patients presenting with suspected or known heart disease to a large and diverse network of centres across Australia providing high-quality health care, this study overcomes some of the limitations of previous research on this topic (eg, data derived from individuals referred to specialist heart care teams and from small cohorts with limited follow-up). Consequently, we demonstrate a high burden of premature mortality associated with all forms of aortic stenosis. This burden imposes a large cost to society in quality-adjusted life years lost.

### Implications of all the available evidence

Though not identifying the specific public health and clinical actions needed to address the burden of aortic stenosis, this study highlights the importance (to clinicians and health administrators alike) of the substantive burden of progressively worse aortic stenosis within our ageing populations. These findings indicate that early detection and recognition of aortic stenosis and further research into mechanisms to slow disease progression might have a profound impact on the longevity and health-care needs of millions of individuals approaching old age around the world.
