# Supplementary material for: Counting the cost of premature mortality with progressively worse aortic stenosis in Australia: a clinical cohort study
Source: Lancet Healthy Longev. Author manuscript; Available in PMC 2022 Sep 19. (PMC9484033; doi:10.1016/S2666-7568(22)00168-4)
Supplement: 1 [file NIHMS1834794-supplement-1.pdf]

THE LANCET
Healthy Longevity

Supplementary appendix

This appendix formed part of the original submission and has been peer reviewed. We post it as supplied by the authors.

Supplement to: Stewart S, Afoakwah C, Chan Y-K, Strom J B, Playford D, Strange GA. Counting the cost of premature mortality with progressively worse aortic stenosis in Australia: a clinical cohort study. *Lancet Healthy Longev* 2022; published online Aug 18. https://doi.org/10.1016/S2666-7568(22)00168-4.

Supplementary Information

Counting the Cost of Premature Mortality with Progressively Worse Aortic Stenosis: A Clinical Cohort Study

Simon Stewart, Clifford Afoakwah, Yih-Kai Chan, Jordan B. Strom, David Playford, Geoffrey A. Strange

This PDF file includes:

Supplementary Figure S1……………………………………………………………………………....2

Supplementary Figure S2……………………………………………………………………………....3

Supplementary Table S1………………………………………………………………………………4

Supplementary Figure S1

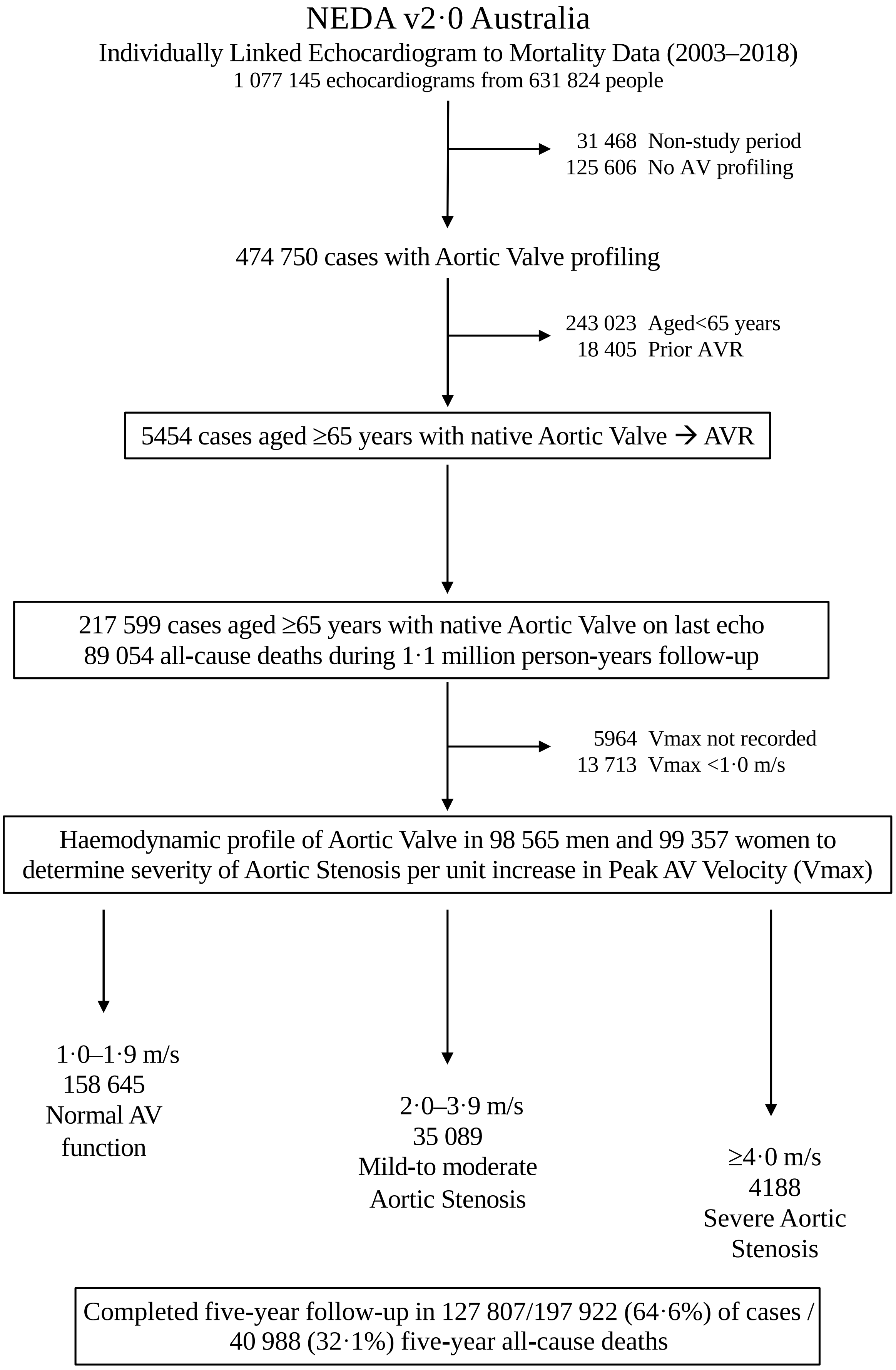

Supplementary Figure S2

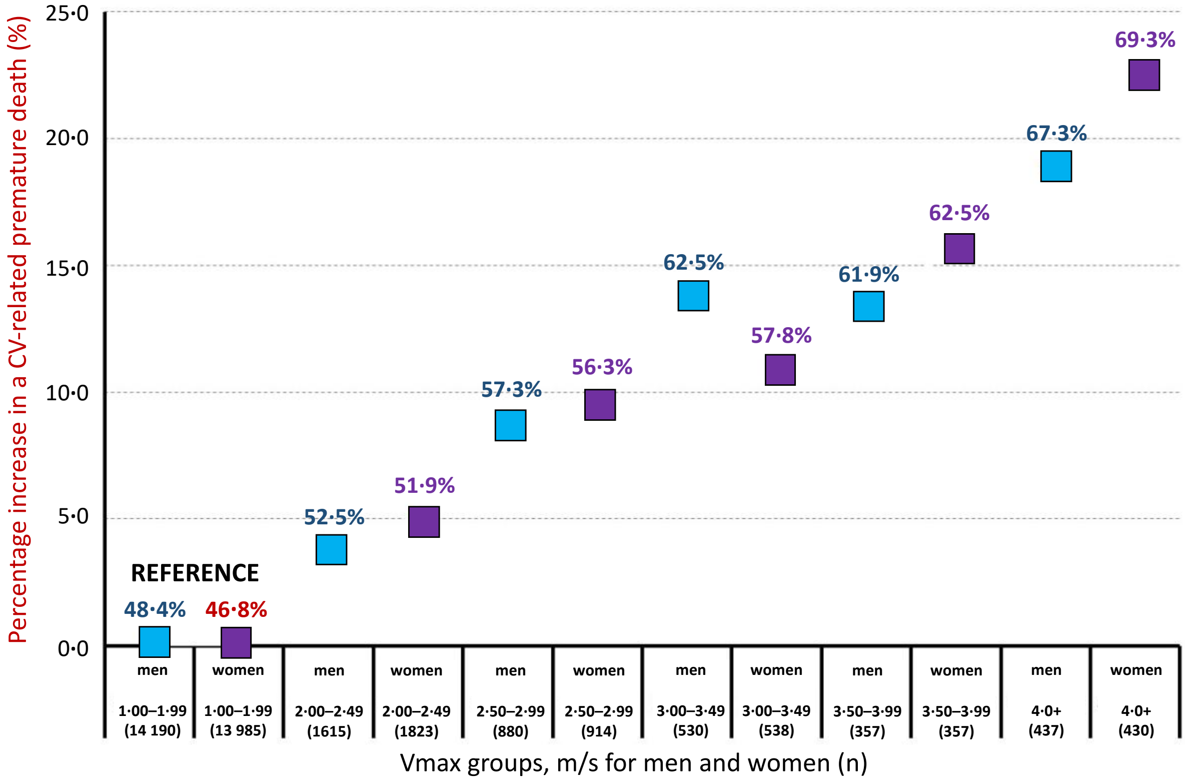

Legend: This figure shows the additional proportion of premature deaths (occurring at any stage of study follow-up) that were associated with a primary or secondary coding of cardiovascular disease as a cause of death with increasing Vmax, relative to those men (blue squares) and women (purple squares) who had normal aortic valve function.

Supplementary Table S1

Vmax Level (m/s)	Sex	YLL Per person	QoL Utility Score	QALYs	Value to short-term QALY (A$)	Value to Long-term QALY (A$)
**Base estimates**						
1·0—1·99	men	6·6	0·71	4·7	201 676	321 811
1·0—1·99	women	7·6	0·71	5·4	232 233	370 570
2·0—2·49	men	5·9	0·71	4·2	180 286	287 680
2·0—2·49	women	7·4	0·71	5·3	226 122	360 818
2·50—2·99	men	5·7	0·71	4·0	174 175	277 928
2·50—2·99	women	6·9	0·71	4·9	210 843	336 439
3·0—3·49	men	5·4	0·71	3·8	165 008	263 300
3·0—3·49	women	6·7	0·71	4·8	204 732	326 687
3·50—3·99	men	5·6	0·71	4·0	171 119	273 052
3·50—3·99	women	6·3	0·71	4·5	192 509	307 183
4·0+	men	5·6	0·71	4·0	171 119	273 052
4·0+	women	5·5	0·71	3·9	168 063	268 176
**Upper–bound estimates**						
1·0–1·99	men	6·6	0·79	5·2	224 400	358 071
1·0–1·99	women	7·6	0·79	6·0	258 400	412 325
2·0– 2·49	men	5·9	0·79	4·7	200 600	320 094
2·0– 2·49	women	7·4	0·79	5·8	251 600	401 474
2·50–2·99	men	5·7	0·79	4·5	193 800	309 244
2·50–2·99	women	6·9	0·79	5·5	234 600	374 347
3·0–3·49	men	5·4	0·79	4·3	183 600	292 968
3·0–3·49	women	6·7	0·79	5·3	227 800	363 497
3·50–3·99	men	5·6	0·79	4·4	190 400	303 818
3·50–3·99	women	6·3	0·79	5·0	214 200	341 795
4·0+	men	5·6	0·71	4·0	171 119	273 052
4·0+	women	5·5	0·71	3·9	168 063	268 176
**Lower–bound estimates**						
1·0–1·99	men	6·6	0·55	3·6	155 376	247 930
1·0–1·99	women	7·6	0·55	4·2	178 918	285 496
2·0– 2·49	men	5·9	0·55	3·2	138 897	221 635
2·0– 2·49	women	7·4	0·55	4·0	174 209	277 983
2·50–2·99	men	5·7	0·55	3·1	134 188	214 122
2·50–2·99	women	6·9	0·55	3·8	162 438	259 200
3·0–3·49	men	5·4	0·55	3·0	127 126	202 852
3·0–3·49	women	6·7	0·55	3·7	157 730	251 687
3·50–3·99	men	5·6	0·55	3·1	131 834	210 365
3·50–3·99	women	6·3	0·55	3·4	148 313	236 661
4·0+	men	5·6	0·48	2·7	115 204	183 829
4·0+	women	5·5	0·48	2·6	113 147	180 547
